# Supplementary material for: Predicting increases in COVID-19 incidence to identify locations for targeted testing in West Virginia: A machine learning enhanced approach
Source: PLoS One. 2021 Nov 3;16(11):e0259538. doi: 10.1371/journal.pone.0259538 (PMC8565789; doi:10.1371/journal.pone.0259538)
Supplement: S1 Appendix — (PDF) [file pone.0259538.s001.pdf]

Distribution and expectation of daily incidence

The daily incidence has a Poisson distribution with parameter $\Lambda_t R_t$. R_t is represented as a random variable following a gamma distribution with parameters a, b:

$$P(k|R_t, \Lambda_t) = \frac{(\Lambda_t R_t)^k}{k!} e^{-\Lambda_t R_t}$$

$$f(R_t|a, b) = \frac{1}{b^a \Gamma(a)} R^{a-1} e^{-\frac{R}{b}}$$

where $\Gamma(a)$ is the usual Gamma function defined as:

$\Gamma(z) = \int_0^\infty t^{z-1} e^{-t}\, dt \rightarrow \Gamma(n+1) = n!$ (if n is a positive integer)

$$\Gamma(z+1) = z\Gamma(z)$$

Denote by $C_{a,b}$ the normalization constant for the Gamma distribution:

$$\frac{1}{C_{a,b}} = \int_0^\infty dR\, R^{a-1} e^{-\frac{R}{b}} = b^a \int_0^\infty du\, u^{a-1} e^{-u} = b^a \Gamma(a)$$

$$C_{a,b} = \frac{1}{b^a \Gamma(a)}$$

$$C_{a+1,b} = \frac{1}{b^{a+1}\Gamma(a+1)} = \frac{1}{ab} C_{a,b}$$

The PMF of the expected number of cases is obtained by integrating over the values of R_t:

$$P(k|\Lambda_t, a, b) = \int_0^\infty dR \frac{(\Lambda_t R)^k}{k!} e^{-\Lambda_t R} \cdot C_{a,b} \cdot R^{a-1} e^{-\frac{R}{b}}$$

The integrand is proportional to a gamma distribution with parameters $a' = a + k,\ \frac{1}{b'} = \frac{1}{b} + \Lambda_t$

$$P(k) = \frac{\Lambda_t^k C_{a,b}}{k!} \int_0^\infty dR\, R^{k+a-1} e^{-R\left(\Lambda_t + \frac{1}{b}\right)} = C_{a,b} \frac{\Lambda_t^k}{k!} \frac{\Gamma(a+k)}{\left(\Lambda_t + \frac{1}{b}\right)^{a+k}} = \frac{(\Lambda_t b)^k}{(\Lambda_t b + 1)^{a+k}} \cdot \frac{\Gamma(a+k)}{k!\,\Gamma(a)}$$

Or (use $\Gamma(z+1) = z\Gamma(z)$)

$$P(k|a, b) = \frac{1}{(b\Lambda_t + 1)^a} \left(\frac{b\Lambda_t}{b\Lambda_t + 1}\right)^k \prod_{j=1}^{k} \frac{(a+j)}{j}$$

The expected number of new infections follows from working out the Gamma-Poisson distribution and coincides with the infection potential multiplied by the expected R

$$\langle I_t \rangle = \Lambda_t R_t \rightarrow \big\langle \langle I_t \rangle (R_t) \big\rangle_{R_t} = \Lambda_t \langle R_t \rangle = \Lambda_t ab$$

$$\langle I \rangle = \sum_{k=1}^{\infty} k \int_0^\infty dR \frac{(\Lambda_t R)^k}{k!} e^{-\Lambda_t R} \cdot C_{a,b} \cdot R^{a-1} e^{-\frac{R}{b}} = \int_0^\infty dR\, \Lambda_t R \sum_{\ell=0}^{\infty} \frac{(\Lambda_t R)^\ell}{\ell!} e^{-\Lambda_t R} \cdot C_{a,b} \cdot R^{a-1} e^{-\frac{R}{b}} = \Lambda_t C_{a,b} \int_0^\infty dR \cdot R^a e^{-\frac{R}{b}}$$
